# Genetic Relationship, Virulence Factors, Drug Resistance Profile and Biofilm Formation Ability of *Vibrio parahaemolyticus* Isolated From Mussel

**DOI:** 10.3389/fmicb.2019.00513

**Published:** 2019-03-20

**Authors:** Md. Ashrafudoulla, Md. Furkanur Rahaman Mizan, Heedae Park, Kye-Hwan Byun, Nari Lee, Si Hong Park, Sang-Do Ha

**Affiliations:** ^1^Department of Food Science and Technology, Advanced Food Safety Research Group, Brain Korea 21 Plus, Chung-Ang University, Anseong, South Korea; ^2^Food Safety Research Group, Korea Food Research Institute, Seongnam, South Korea; ^3^Food Science and Technology, Oregon State University, Corvallis, OR, United States

**Keywords:** *Vibrio parahaemolyticus*, seafood, genetic relationship, antibiotic resistance, biofilm

## Abstract

The objective of this study was to investigate the virulence factors, genetic relationship, antibiotic resistance profile and the biofilm formation ability of *Vibrio parahaemolyticus* isolates on shrimp and mussel surfaces at 30°C. In this study, eight (*n* = 8) *V. parahaemolyticus* isolated from mussel were examined. We used the polymerase chain reaction (PCR) to examine the distribution of different genes, and Repetitive Extragenic Palindromic-PCR (REP-PCR) to compare the genetic relationship. Disk diffusion technique was used to assess antibiotic and multiple-antibiotic resistance. The biofilm formation assay, and field emission scanning electron microscopy (FE-SEM) were used to evaluate biofilm formation ability. Transmission Electron Microscope (TEM) was used to observe the morphological structure of bacterial cell. Our results indicated that the biofilm-associated genes, 16S rRNA, *toxR*, and *tdh*, were present in all the tested *V. parahaemolyticus* isolates (*n* = 8). Approximately, 62.5% (5 isolates among 8 isolates) isolates showed strong multiple-antibiotic resistance index with an average value of 0.56. All isolates (*n* = 8) showed strong genetic relationship and significant biofilm formation ability on shrimp and mussel surfaces. This study demonstrated that the presence of virulence factors, high multiple antibiotic resistance index (MARI) values, and effective biofilm formation ability of *V. parahaemolyticus* isolates could be a great threat to human health and economic values in future. It was also suggested that a high resistance rate to antibiotic could be ineffective for treating *V. parahaemolyticus* infections. The continuous monitoring of *V. parahaemolyticus* antibiotic, molecular and biofilm characteristics is needed to increase seafood safety.

## Introduction

Seafood is recognized as a nutritious and healthy food choice, and is accepted by increasing numbers of consumers worldwide (Hellberg et al., 2012). Every year, above 100.2 million metric tons of seafood are caught and consumed in the world (Cisneros-Montemayor et al., 2016). In 2014, the value of imported seafood in Korea, China, and the United States were 4.16, 1.12 billion, and 222 million, respectively (Mizan et al., 2018). However, the main obstacles in the consumption of seafood are their high perishability and health risk due to contamination by pathogens (Reyhanath and Kutty, 2014). Therefore, seafood safety is considered as necessary to maintain public health and seafood processing (Jahan, 2012; Machado and Gram, 2015).

In the aquaculture industry, black tiger shrimp (*Penaeus monodon*) plays an important role in the economic aspect and cultured in both inland and marine (Szuster, 2006). *Vibrio parahaemolyticus* is the most prevalent shrimp pathogen encountered in aquaculture, causes in shrimp *Vibrio*sis with the potential for severe health crisis (Mohammad et al., 2005; Kleter et al., 2009; Sani et al., 2013; Zhang et al., 2014). In China, shrimp contaminated with *V. parahaemolyticus* has been accompanied with outbreaks of food borne illnesses (Peng et al., 2010). In Australia two outbreaks of gastroenteritis occurred between 1990 and 1992 due to the consumption of *V. parahaemolyticus* contaminated cooked shrimps imported from Indonesia (Sumner, 2011). The mussel is valued worldwide for its sensory and nutritional qualities. Spain is the main supplier of mussel to the European market, coming 98% of this production from Galicia (Garrido-Maestu et al., 2016). During 1997 to 2010, Global production of mussels has increased up to 1.9 million tons worldwide. This represented 95% of the world mussel production, in comparison to 83% in 1997 (Ferreira et al., 2014). In recent years, Galicia has been recognized as the largest producers of mussels, accounting for the 15 to 25% of the world’s annual mussel production (Miguez et al., 2009; Costas-Rodríguez et al., 2010; Caballero-Miguez et al., 2012). But several studies have demonstrated the presence of pathogenic species of the genus *Vibrio* in the Galician Rias (Lozano-León et al., 2003; Martinez-Urtaza et al., 2004, 2008; Rodriguez-Castro et al., 2010). As mussel is a good vehicle for *Vibrio* species, *V. parahaemolyticus* can survive in mussel with potential contamination (Mannas et al., 2014). Therefore, *V. parahaemolyticus* constitute a potential risk to consumers for having improperly processed shellfish (FDA BAM, 2004). Several post-harvest processes, including low-temperature pasteurization and irradiation have been developed for reducing *Vibrio*s in aquaculture but they are expensive (Chae et al., 2009).

*Vibrio* infections occurred due to the presence of virulence factor. The strains of *V. parahaemolyticus* contain virulence factor, including adhesins (Type I pilus), *toxR*, biofilm, thermostable direct hemolysin (*tdh*), TDH-related hemolysin (*TRH*) encoded by *trh* gene, VPaI-2, VPaI-3, VPaI-6, type III secretion systems (T3SS), and type VI secretion systems (T6SS) (Chao et al., 2009, 2010; Broberg et al., 2011; Salomon et al., 2013: Letchumanan et al., 2014). In the United States, more than 80% of gastroenteritis and 90% of septicemia infections happened during 1988 to 1997, due to the consumption of oysters (Daniels and Shafaie, 2000). It was also reported in 2006 that *V. parahaemolyticus* was responsible for 177 cases due to having raw shellfish in the United States (Yoon et al., 2008). Therefore the consumption of *V. parahaemolyticus* contaminated seafood is one of the greatest source of infection in America as well as in Asia (Hongping et al., 2011).

The other problem associated with *V. parahaemolyticus* is due to the prevalence of antibiotic resistance in aquaculture. The extensive use of antibiotics for the treatment of infections caused by *V. parahaemolyticus*, has increased the incidence of antibiotic-resistant strains (Cabello et al., 2013; Yano et al., 2014; Letchumanan et al., 2015; Xie et al., 2017: Yang Y. et al., 2017; Lee et al., 2018). *V. parahaemolyticus* has shown resistant property against numerous antibiotics including ampicillin, ciprofloxacin, cephazolin, streptomycin, cefotaxime, and cefuroxime sodium (Al-Othrubi et al., 2014; Jiang et al., 2014; Sudha et al., 2014; Yano et al., 2014). The multidrug resistance of *V. parahaemolyticus* is also increasing gradually due to the excessive use of antibiotics in the fields of agriculture and aquaculture (Kang et al., 2017). Antibiotic-resistant bacteria may represent a potential threat to human health via direct transmission through the food chain or the transfer of resistance genes to other human (Duran and Marshall, 2005; Guglielmetti et al., 2009; Ma et al., 2018). In aquaculture farming, an appropriate policy is essential for using antibiotics (Odeyemi and Stratev, 2016). Moreover, the monitoring of antibiotic resistance patterns of *V. parahaemolyticus* in seafood is also important (Odeyemi and Stratev, 2016), because it is a high concern for human health (Xie et al., 2017). This emerging incidence of antibiotic resistance in *V. parahaemolyticus* has generated a growing interest in identifying new strategies for preventing infections related to *V. parahaemolyticus* biofilms (Su and Liu, 2007; Lopatek et al., 2018; Silva et al., 2018; Jiang et al., 2019).

Bacterial biofilms are architecturally complex assemblies of microorganisms that adhere to biotic or abiotic surfaces and are attached within a matrix of extracellular polymeric substances (Costerton et al., 1999; Hall-Stoodley et al., 2004; Flemming and Wingender, 2010; Mizan et al., 2015). Bacteria in biofilms can be 1,000-fold more resistant to environmental stress than planktonic cells are (Brooun et al., 2000). *V. parahaemolyticus* is able to produce adherence factors, to adhere to surfaces for biofilm formation (Donlan, 2002). Biofilm cells are more resistant to disinfectants and antibacterial agents than the same bacteria in a free-swimming state, so the environmental survival, infectivity and transmission are enhanced due to strong biofilm formation ability of this pathogen (Kadam et al., 2013; Elexson et al., 2014a). In our knowledge, this is the first study to check the biofilm formation ability of *V. parahaemolyticus* isolates on mussel surface.

Based on the epidemiological importance of *V. parahaemolyticus* and the concern of eating undercooked shellfish, the present study was design to verify the genetic relationship, virulence factors, antibiotic resistance profile and biofilm formation ability of *V. parahaemolyticus* isolated from mussel from the west coast area of Korea.

## Materials and Methods

### Bacterial Strains, Growth Conditions, and Preparation of Bacterial Suspensions

A total of 10 *V. parahaemolyticus* strains, were included in this study. Among 10 *V. parahaemolyticus* strains, two (*n* = 2) were reference strains (ATCC17802 and ATCC27969), and eight (*n* = 8) were environmental isolates (Table 1) from sea mussel. These environmental isolates were obtained from the National Institute of Fisheries Science (NIFS), South Korea. *V. parahaemolyticus* isolates were elementary identified as blue-green colored colonies using thiosulfate citrate bile salts sucrose agar (TCBSA, Difco Laboratories, Sparks, MD, United States). Isolates were transferred onto nutrient agar (Difco Laboratories) and cultivated at 30°C for 24 h. Biochemical tests were directed using a VITEK 2 compact system (bioMerieux, Grenoble, France) to assure the phenotypical identity of the isolates. Prior to each experiment, the strains were activated by transferring from stocks which stored at -80°C to CHROMagar *Vibrio* plates (CHROMagar, Paris, France) and incubated overnight at 30°C. After 18–24 h incubation a single colony was taken from each plate and inoculated into 5 ml Luria-Bertani (LB) broth (2% NaCl; Difco Laboratories), and then incubated overnight at 30°C in a shaking incubator (Vision Scientific, VS-8480, South Korea) at 220 rpm. Subsequently, the *V. parahaemolyticus* cultures were centrifuged (11,000 × *g* at 4°C for 10 min), and then washed and resuspended in peptone water (PW; BD diagnostics, Franklin Lakes, NJ, United States), and made a target concentration (CFU/ml) for the final experiment.

**Table 1:** The information about regions, season, sources, and water temperature of *V. parahaemolyticus* isolates used in this study.

Isolates	Source of isolation	Area (Island)	Latitude/longitude	Date Year, 2016	Water Temp. (°C)
NIFS18	Sea mussel	Seungbong	34°55′26′′N/128°26′24′′E	05, September,	25.67
NIFS24	Sea mussel	Seungbong	34°55′20′′N/ 128°30′04′′E	06, September,	26.28
NIFS25	Sea mussel	Daeijak	34°51′35′′N/ 128°04′29′′E	18, October	22.19
NIFS26	Sea mussel	Soijak	34°55′33′′N/127°57′17′′E	26, October	19.57
NIFS27	Sea mussel	Daeijak	34°50′25′′N /128°05′06′′E	18, October	22.01
NIFS28	Sea mussel	Soya	34°44′21′′N/127°29′13′′E	25, October	17.20
NIFS29	Sea mussel	Soya	34°28′29′′N/ 127°26′18′′E	12, October	22.20
NIFS30	Sea mussel	Daeijak	34°55′33′′N /127°57′17′′E	26, October	19.57

*Temp. = Temperature; NIFS = National Institute of Fisheries Science.*

### Polymerase Chain Reaction

In the present study, a single polymerase chain reaction (PCR) assay was performed to test virulence factor. The nineteen oligonucleotide primer pairs (Table 2) were considered to evaluate the presence and absence of one specific primer for 16S rRNA, two pandemic clone genes, one *toxR* gene, TDH-related hemolysin *trh* gene, three biofilm genes, two VPaI-2 open reading frames (ORFs), one VPaI-3 ORFs, two VPaI-6 ORFs, two T6SS genes, two type I pilus genes, and two T3SS1 genes. All of the primers selected in this study were synthesized by the Bioneer Corporation (Daejeon, South Korea). The Tissue Kit and DNeasy Blood (QIAGEN, Venlo, Netherlands) were used to purify total DNA according to the instructions of manufacturer. The PCR reactions (25 μl) contained 14 μl of PCR mix (Solutions for Genetic Technologies, Daejeon, South Korea), 2 μl of each of the primers (10 μM), 2 μl of the DNA template, and milli-Q water. The reactions were as follows: an initial denaturation step at 95°C for 3 min, 40 cycles of 95°C for 30 s, 40–57°C (depending on product annealing temperature) for 30 s, and 72°C for 1 min, followed by a final incubation at 72°C for 5 min. PCR amplification was performed in triplicate for genomic DNA from each of the strains. The products were identified using electrophoresis on a 1.5% agarose gel and Safe View Classic staining (0.008%, v/v) (Applied Biological Materials Inc., Richmond, Canada). A 100-bp ladder (BioFACT, Daejeon, South Korea) was selected as the molecular weight marker.

**Table 2:** The primers used in the current study.

Primer	Sequence (5′ to 3′)	Target gene	Amplicon size (bp)	Reference
VparaF VparaR	GCTGACAAAACAACAATTTATTGTT	16S rRNA	170	Rojas et al., 2011
	GGAGTTTCGAGTTGATGAAC			
*toxRS*/old-F *toxRS*/old-R	TAATGAGGTAGAAACG	*toxRS* sequence of theold O3:K6 clone	651	Okura et al., 2003
	ACGTAACGGGCCTACG			
*toxR*-F *toxR*-R	GTCTTCTGACGCAATCGTTG	*toxR*	368	Kim et al., 1999
	ATACGAGTGGTTGCTGTCATG			
L-*tdh* R-*tdh*	GTAAAGGTCTCTGACTTTTGGAC	Thermostable direct hemolysin	269	Nishibuchi and Kaper, 1995
	TGGAATAGAACCTTCATCTTCACC			
L-*trh* R-*trh*	TTGGCTTCGATATTTTCAGTATCT	TDR-related hemolysin (*TRH*)	500	Rojas et al., 2011 Silva et al., 2018
	CATAACAAACATATGCCCATTTCCG			
VP0950-F VP0950-R	GCCAAACTTCTCAAACAACA	Biofilm	298	Chao et al., 2010
	ATGAAACGCAATTTACCATC			
VP0952-F VP0952-R	TATGATGGTGTTTGGTGC		276	
	TGTTTTTCTGAGCGTTTC			
VP0962-F VP0962-R	GACCAAGACCCAGTGAGA		358	
	GGTAAAGCCAGCAAAGTT			
VP0634-F VP0634-R	AGATGTCTTTGTTCACCCT	VPaI-2	473	Chao et al., 2010
	CGAAGTCGGCTTTGTAGTT			
VP0636-F VP0636-R	TGAAAGTGACGGCTCCAATC	VpaI-2	207	
	CTGCGTTCAGTTCCACATCG			
VP1094-F VP1094-R	GATTCAAGGTGGATTTCG	VpaI-3	219	
	ATAAGCGGGTTCTTCGTC			
VP1253-F VP1253-R	GTCCCTCAATCTGTGCTT	VpaI-6	898	
	GCTGACAATCTTCGCTCT			
VP1263-F VP1263-R	TCGTGGACAACTATGAAGC	VpaI-6	293	
	AAGTAGGAACTGACGGAAAC			
VP1409-F VP1409-R	TGTTGCTTTCTATTGCGAC	T6SS	869	
	CCATAACGACTTTTCTTTC			
VP1418-F VP1418-R	AAACCAGCCTCAGCAACAAG	T6SS	308	
	TAATAGCGGCGATAAATCCA			
VP1510-F VP1510-R	TTCAGGTTTCAGGGTTC	Type I pilus	511	
	GCTTGCTCATAGTTGGC			
VP1506-F VP1506-R	CCGAACATTTAGAAGGC	Type I pilus	399	
	AGCGAGAAAGCAGAACA			
VP1677-F VP1677-R	TAGTCAGATAGCAACCAACA	T3SS1	548	Chao et al., 2009
	CATCAGCGAAATGAGAAACA			
VP1690-F VP1690-R	CACCAATGTGAGCCAAAAAG	T3SS1	384	
	ATAAACACCGATGCCGAAGC			

### Repetitive Extragenic Palindromic-PCR (REP-PCR)

REP-PCR, used for chromosomal comparisons of *V. parahaemolyticus* isolates, was conducted using two primers: REP-1D, 5’-NNN RCG YCG NCA TCM GGC-3’; and REP-2D, 5’-RCG YCT TAT CMG GCC TAC-3’ (where M is A or C, R is A or G, Y is C or T, and N is any nucleotide) as reported previously (Wong and Lin, 2001). The experiment was performed followed by Mizan et al. (2017), and a digital image was captured through a charge coupled device camera (Gel Doc XR system, Bio-Rad). The resulting fingerprints were analyzed using FPQuest software (Bio-Rad Laboratories, Inc., Hercules, CA, United States). Similarities between digitized profiles were counted using Pearson’s correlation, and an average linkage (unweighted pair group method with arithmetic mean, UPGMA) dendrogram was obtained.

### Antibiotic Susceptibility Testing

The antibiotic susceptibility of *V. parahaemolyticus* isolates was determined using the disk diffusion technique (NCCLS, 2003). For testing antibiotic susceptibility, selective media were used with slight modifications as previously described (Temmerman et al., 2003; McLain et al., 2016; Vandeplassche et al., 2017). Eleven antibiotics were tested in this study (Table 3). The 10 *V. parahaemolyticus* isolates were spread on CHROMagar plates onto which antibiotic disks were then placed. The plates were incubated at 30°C for 18–24 h under aerobic conditions. The zones of inhibition were measured according to the guidelines of Clinical and Laboratory Standards Institute (CLSI, 2010). The multiple-antibiotic resistance (MAR) index of the isolates was defined as *x*/*y*, where *x* represents the number of antibiotics to which a particular isolate was resistant, and *y* represents the number of antibiotics to which the isolate was susceptible (Krumperman, 1983). Tetracycline and ciprofloxacin are recommended antibiotics to treat *V. parahaemolyticus* illnesses (Park et al., 2018). Previous reports performed in South Korea showed the antibiotic susceptibility profile of *V. parahaemolyticus* isolates in seawater samples and found that 3.0–12.2% of isolates were resistant to tetracycline and ciprofloxacin, respectively (Son et al., 2005; Kim et al., 2014). Other studies reported using different antibiotics against *V. parahaemolyticus* for detecting antibiotic susceptibility (Han et al., 2012; Yang J. H. et al., 2017; Park et al., 2018). Most of the antimicrobials tested in this study are using in agriculture and aquaculture fields (Kim S. et al., 2016; Kang et al., 2017), as well as in the treatment of *vibrio* infections (Shaw et al., 2014).

**Table 3:** Antibiotics used in this study.

Serial number	Antibiotic name	Reference
01	Erythromycin (E; 15 μg)	Kang et al., 2017
02	Vancomycin (VA; 30 μg)	
03	Kanamycin (K; 30 μg)	
04	Chloramphenicol (C; 30 μg)	
05	Streptomycin (S; 10 μg)	
06	Ampicillin (AM; 10 μg)	
07	Ciprofloxacin (CIP; 5 μg)	
08	Gentamicin (GM; 10 μg)	
09	Tetracyclin (TE; 30 μg)	
10	Clindamycin (CC; 2 μg),)	García-Hernández et al., 2016
11	Penicillin (G; 10 μg)	Tan et al., 2017

### Preparation of Inoculum for Food Samples

The cultures in LB containing 2% NaCl were centrifuged (10,000g for 12 min at 4°C) and the pellets were washed with sterile phosphate-buffered saline (PBS, pH 7.2). The pellets were resuspended in the same amount of PBS. These inocula were used to form biofilm on shrimp and mussel surfaces.

### Preparation of Shrimp and Mussel Surfaces, Biofilm Formation, and Detachment Population

Black tiger shrimp (*P. monodon*) and mussel (*Mytilus coruscus*) were purchased from a native grocery store in Anseong, South Korea. Surface preparation, biofilm formation, and detachment procedure were performed followed by Han et al. (2016) with minor modifications. Using a scalpel, the shrimp head surface and mussel cover surface were aseptically cut into 2 × 2 cm^2^ that were then washed with sterile distilled water to remove the flesh. Prior to inoculation with *V. parahaemolyticus*, the surfaces were placed in an open sterile petri dish and subjected to ultraviolet-C treatment for 30 min on each side to minimize the background flora. Preliminary experiments confirmed that a UV-C treatment time of 30 min was sufficient to remove the background microbiota below the detection level cultured on Trypticase Soy Agar (TSA) plates (Han et al., 2016; Jahid et al., 2019). The incubated bacterial cultures were centrifuged (10,000 × *g* at 4°C for 12 min). The resulting pellets were washed three times using PBS, and resuspended into LB to attain the final concentration of bacterial cells (10^5^ CFU/ml), and then used to form biofilm on shrimp and mussel surfaces. Each surface was completely submerged into 10 ml LB in 50-ml Falcon tubes (SPL Life Sciences Co., Ltd., Pocheon, South Korea). Each isolate (10^5^ CFU/ml) was added to a Falcon tube and incubated for 24 h without shaking. After biofilm formation, the shrimp and mussel surfaces were removed from the Falcon tube and washed at least three times with PBS to remove planktonic bacteria, and transferred into a sterile stomacher bag containing 10 ml peptone water (PW; BD Diagnostics, Franklin Lakes, NJ, United States), and processed using a Stomacher (Bag Mixer; Interscience, Saint Nom, France) at the highest speed of 4 for 2 min to release the biofilm-forming bacteria cells from shrimp and mussel surfaces. Enumeration of each *V. parahaemolyticus* isolate was obtained by serial dilutions and spread onto CHROMagar *Vibrio* plates. After incubation at 30°C for 24 h, the resulting colonies were counted and expressed as CFU/cm^2^ for the biofilm populations. *V. parahaemolyticus* can contaminate both shrimp (Devi et al., 2009; Jun et al., 2012; Saifedden et al., 2016; Ahmed et al., 2018), and mussel (Bauer et al., 2006; Rojas et al., 2011; Jun et al., 2012; Lopatek et al., 2015). Previous studies reported that the *V. parahaemolyticus* isolates, collected from different sources (shrimp, crab, oysters and mussels) can generate biofilm on other different surfaces (Ahmed et al., 2018; Fang et al., 2018; Rosa et al., 2018), and it was already reported that *V. parahaemolyticus* can make biofilm on shrimp surface (Mizan et al., 2018). We considered shrimp as a tested surface along with a mussel though strains were isolated from mussel in this study.

### Examination of Biofilms via Field Emission Scanning Electron Microscopy (FE-SEM)

The biofilm formation ability of representative isolates (ATCC27969 and NIFS29) were examined by FE-SEM. The surfaces were prepared with some modifications as described previously by Jahid et al. (2013). The surfaces were fixed at room temperature for 4 h with 2.5% glutaraldehyde. The surfaces were then serially treated with ethanol (50% for 15 min, 60% for 15 min, 70% for 15 min, 80% for 15 min, 90% for 15 min, and twice with 100% for 15 min each time) and successively dehydrated by soaking in 33, 50, 66, and 100% hexamethyldisilazane in ethanol for 15 min each time. The dehydrated surfaces were sputter coated with platinum and visualized by FE-SEM (Hitachi/Baltec, S-4700). In this study, NIFS29 was selected as a representative bacterium among all isolates to present our data. NIFS29 was selected based on the higher biofilm formation ability. However, the biofilm formation ability between NIFS28 and NIFS29 were not significantly different.

### Transmission Electron Microscope (TEM) Sample Preparation and Imaging

A TEM sample was prepared with minor modifications from the previous study (Golding et al., 2016). The sample was adsorbed for 1 min to a formvar film on a carbon-coated 400-mesh copper grid. It was then washed 3 times in distilled water and negatively contrasted with 2% methylamine tungstate (Nano-W; Nanoprobes, Yaphank, NY, United States). The image was taken at 200 kV using a FEI Tecnai 20 transmission electron microscope (FEI Company, Hillsboro, OR, United States). The magnifications of 3,500× to 19,000× was considered for TEM images.

### Statistical Analysis

Each experiment was performed independently in triplicate. The data were expressed as mean ± standard error (SE). Data were analyzed using Microsoft excel and Duncan’s new multiple tests with SAS software (version 9.2, SAS Institute Inc., Cary, NC, United States). *P*-values < 0.05 were considered significantly different.

## Results

### Gene Detection of *V. parahaemolyticus*

The distribution of specific and virulence-associated genes was represented in Table 4. The *V. parahaemolyticus* isolates shown positive PCR amplification to specific genes: (16S rRNA), marker (*toxR*), pathogenic gene (L-*tdh*), biofilm genes VP950 (encoding a lipoprotein-related protein), VP952, and VP962 (encoding a hypothetical protein), type VI secretion T6SS (VP1409), Type I pilus (VP1510), pathogenicity island-2 (VPaI-2), and VPaI-6 (VP1253). Our results also indicated that 87.5% (*n* = 8) of *V. parahaemolyticus* isolates harbored the complete type three secretion T3SS (VP1690), tox-RS/Old, and VPaI-6 (VP1263); 50% (*n* = 8) of isolates harbored the complete Type I pilus (VP1506) and type VI secretion T6SS (VP1418) genes. For *trh* gene, all isolates (*n* = 8) shown negative amplification of PCR.

**Table 4:** Detection of genes involved in biofilm formation as well as the pathogenicity of *V. parahaemoliticus* isolates.

*V. parahae-moliticus* isolates	16S rRNA	toxR	tox-RS/ Old	L-tdh	*trh*	VP 0950	VP 0952	VP 0962	VPaI-2 VP0634	VPaI-2 VP0636	VPal-3 VP1094	VPaI-6 VP1263	VPaI-6 VP1253	T6SS VP1409	T6SS VP1418	VP1506 Type 1 pilus	VP1510 Type I pilus	T3SS VP1690	T3SS VP1677
ATCC17802	+	+	+	+	+	+	+	+	+	+	+	+	+	+	+	–	+	+	–
ATCC27969	+	+	+	+	–	+	+	+	+	+	+	+	+	+	+	–	+	+	–
NIFS18	+	+	+	+	–	+	+	+	+	+	+	+	+	+	+	–	+	+	+
NIFS24	+	+	+	+	–	+	+	+	+	+	+	+	+	+	–	–	+	+	+
NIFS25	+	+	+	+	–	+	+	+	+	+	+	+	+	+	–	+	+	–	+
NIFS26	+	+	+	+	–	+	+	+	+	+	+	+	+	+	–	+	+	+	+
NIFS27	+	+	–	+	–	+	+	+	+	+	+	+	+	+	–	+	+	+	+
NIFS28	+	+	+	+	–	+	+	+	+	+	+	+	+	+	+	+	+	+	+
NIFS29	+	+	+	+	–	+	+	+	+	+	+	–	+	+	+	–	+	+	–
NIFS30	+	+	+	+	–	+	+	+	+	+	+	+	+	+	+	–	+	+	–

*+, presence of gene sequence; –, absence of gene sequence.*

### REP-PCR Fingerprinting

**FIGURE 1:**
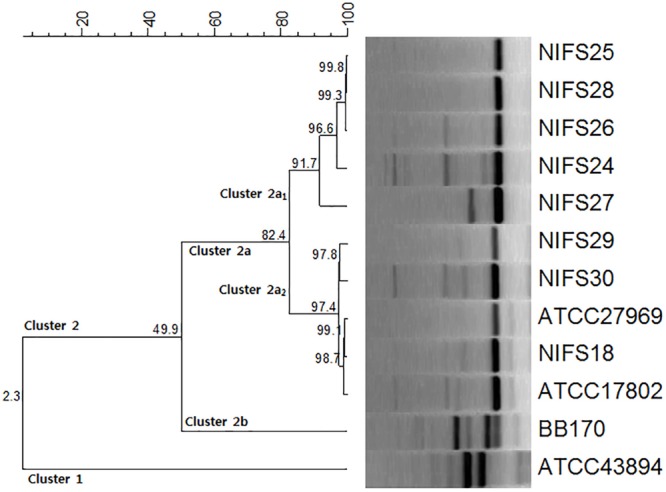
Repetitive extragenic palindromic sequence-based PCR (REP-PCR) results for the 10 *Vibrio parahaemolyticus* isolates and reference strains *Escherichia coli* ATCC43894 and *Vibrio harveyi* BB170.

The genetic relationships among the *V. parahaemolyticus* isolates were analyzed by REP-PCR. All the isolates demonstrated a common band at ∼ 400 bp with amplification ranging from 100 to 2,000 bp. According to the REP-PCR banding pattern, all the isolates were classified into two major clusters, cluster 1 and cluster 2 (Figure 1). Cluster 2 was divided into two sub-clusters (cluster 2a and cluster 2b) having 49.9% similarity. In cluster 2a, two main groups (Cluster 2a_1_ and Cluster 2a_2_) were detected for the *V. parahaemolyticus* isolates. NIFS18, NIFS29, NIFS30, ATCC27969, and ATCC17802 showed 97.4% similarity. The other isolates, NIFS25, NIFS28, NIFS26, NIFS24, and NIFS27, showed 91.7% similarity. ATCC43894 (*Escherichia coli*) and BB170 (*Vibrio harveyi*) were used as negative controls. A similarity level of 2.3% was observed between these strains (Figure 1) independently from the *V. parahaemolyticus* isolates.

### Antibiotic Resistance Profile of *V. parahaemolyticus* Isolates

The antibiotic resistance profile of *V. parahaemolyticus* isolates with respect to 11 antibiotics were shown in Table 5. The majority of isolates were resistant to all the antibiotics. The isolates were highly resistant to vancomycin (100%) and penicillin (87.5%) and showed 75% resistance to clindamycin and tetracycline, and 62.5% resistance to kanamycin. The MAR index values of *V. parahaemolyticus* isolates were summarized in Table 6. The MAR values ranged from 0.18 to 0.82, with an average of 0.56. One (12.5%) isolate exhibited the highest MAR index value of 0.82, showing resistance to nine antibiotics.

**Table 5:** Antibiotics resistance profiles of *Vibrio parahaemolyticus* isolates.

Antibiotics	Concentration (μg)	Number of percentage (%)		
		Sensitive (%)	Intermediate (%)	Resistant (%)
Erythromycin (E)	15	3/8 (37.5)	3/8 (37.5)	2/8 (25)
Vancomycin (VA)	30			8/8 (100)
Kanamycin (K)	30	1/8 (12.5)	2/8 (25)	5/8 (62.5)
Chloramphenicol (C)	30	5/8 (62.5)	3/8 (37.5)	
Streptomycin (S)	10	1/8 (12.5)	3/8 (37.5)	4/8 (50)
Ampicillin (AM)	10	1/8 (12.5)	3/8 (37.5)	4/8 (50)
Ciprofloxacin (CIP)	5	4/8 (50)	1/8 (12.5)	3/8 (37.5)
Gentamicin (GM)	10	1/8 (12.5)	3/8 (37.5)	4/8 (50)
Tetracyclin (TE)	30		2/8 (25)	6/8 (75)
Clindamycin (CC)	2		2/8 (25)	6/8 (75)
Penicillin (G)	10		1/8 (12.5)	7/8 (87.5)

**Table 6:** Multiple antibiotic resistance (MAR) index of *V. parahaemolyticus* isolates.

Resistance pattern	Frequency	Isolate code	Percentage (%)	MAR index
VA, TE	1	NIFS24	12.5	0.18
E, VA, P, CC	1	NIFS26	12.5	0.36
E, VA, P, CC, K, S, AM, TE	1	NIFS30	12.5	0.73
VA, P, K, S, TE	1	NIFS25	12.5	0.45
VA, P, CC, K, S, AM, CIP, GM, TE	1	NIFS27	12.5	0.82
VA, P, CC, S, CIP, GM	1	NIFS29	12.5	0.55
VA, P, CC, K, AM, GM, TE	1	NIFS28	12.5	0.64
VA, P, CC, K, AM, CIP, GM, TE	1	NIFS18	12.5	0.73

### Comparison of Biofilm Formation Ability Between Clinical and Environmental Isolates

In this study, environmental isolates showed a higher biofilm formation ability than clinical isolates. For shrimp surface, NIFS25, NIFS28 and NIFS29 showed 6.72, 6.80, and 6.89 log CFU/cm^2^ biofilm formation, respectively (Table 7). For mussel surface, NIFS25, NIFS28 and NIFS29 also shown strong biofilm formation like 6.25, 6.39, and 6.40 log CFU/cm^2^, respectively (Table 7). On the other hand, clinical isolates ATCC17802 and ATCC27969 showed 5.29 and 5.72 log CFU/cm^2^ biofilm formation, respectively (Table 7). The biofilm-forming ability of the isolates may vary under different environmental stress and isolation source. This study indicated that environmental isolates show a great ability to contaminate shellfish.

**Table 7:** Biofilm cell grown at 30°C for 24 h on shrimp and mussel surface.

Isolates	Shrimp surface	Mussel surface
*V. Parahaemolyticus*	log CFU/cm^2^± SD	log CFU/cm^2^± SD
ATCC17802	5.59 ± 0.27^D^	5.29 ± 0.27^C^
ATCC27969	6.19 ± 0.29^C^	5.72 ± 0.19^BC^
NIFS18	6.24 ± 0.16^BC^	6.04 ± 0.10^BA^
NIFS24	6.52 ± 0.14^BAC^	6.12 ± 0.33^BA^
NIFS25	6.72 ± 0.23^BA^	6.25 ± 0.18^BA^
NIFS26	6.21 ± 0.16^C^	5.91 ± 0.13^BA^
NIFS27	6.29 ± 0.27^BC^	6.06 ± 0.21^BA^
NIFS28	6.80 ± 0.13^A^	6.39 ± 0.09^A^
NIFS29	6.89 ± 0.16^A^	6.40 ± 0.17^A^
NIFS30	6.27 ± 0.16^BC^	6.04 ± 0.35^BA^

*Within each treatment, values marked with the same letter are not significantly different based on Duncan’s multiple-range test (*p* > 0.05). SD = Standard deviation.*

### FE-SEM

**FIGURE 2:**
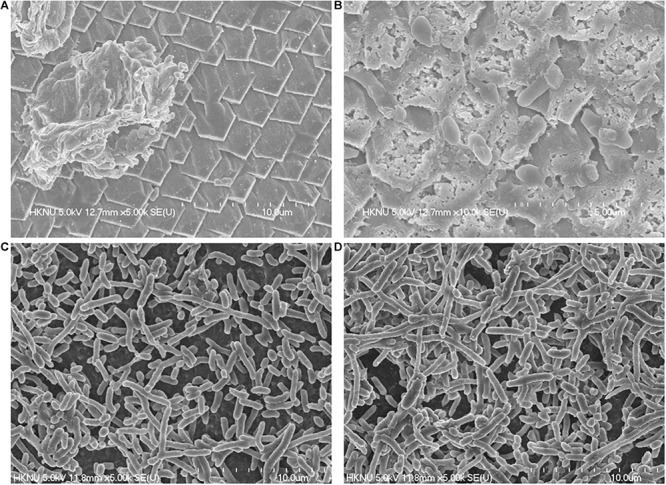
Representative FESEM images of *V. parahaemolyticus* clinical (ATCC27969) and environmental (NIFS29) isolates biofilm formation on mussel and shrimp surfaces. For mussel surface clinical isolate **(A)**, environmental isolates **(B)**; For shrimp surface clinical isolate **(C)**, environmental isolates **(D)**.

The FE-SEM analysis of clinical and environmental isolates were shown in Figure 2. The representative isolates for the clinical and environmental were ATCC27969, and NIFS29, respectively. ATCC27969 and NIFS29 were selected based on their higher biofilm formation ability. In the case of both isolates, biofilms were organized in structure with intact cell-to-cell connections. The morphology of the isolates (ATCC27969 and NIFS29) looked smooth with an intact cell membrane (Figure 2). The environmental isolate showed stronger biofilm formation on both surfaces than on the clinical isolate (Figure 2).

### Morphological Structure Observation

**FIGURE 3:**
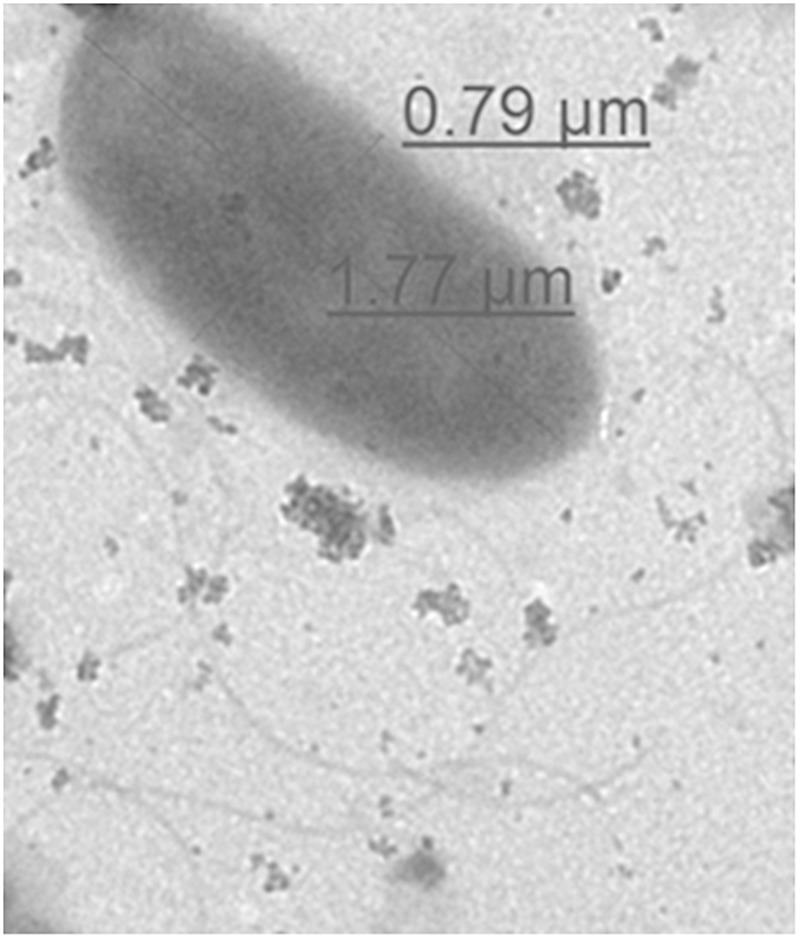
Representative TEM image of *V. parahaemolyticus* environmental isolate cell (NIFS29).

Morphological structure of representative environmental isolate (NIFS29) was observed through TEM. The electron micrographs of NIFS29 cell was displayed in Figure 3. The bacterial cell showed typical character of rod-shaped bacteria. The cell surface was smooth, and the flagella was clear. The diameter of the cell was 0.79 μm in width and 1.77 μm in length (Figure 3), this is within the range of *V. parahaemolyticus* standard diameter (0.5–0.8 μm in width and 1.4–2.4 μm in length)^1^.

## Discussion

Assays based on molecular PCR are useful in detecting the *toxR* gene in *V. parahaemolyticus* isolates (Fabbro et al., 2010). Following detection on the CHROMagar *Vibrio* plates (data not shown), PCR assays for *toxR*, and genes associated with biofilm formation and pathogenicity were conducted for molecular characterization of the *V. parahaemolyticus* isolates. The *toxR* gene fragment (∼368 bp) specific to *V. parahaemolyticus* (Zulkifli et al., 2009) and three biofilm-associated genes were effectively amplified from all the isolates (Table 4); similar results were reported in another study (Mizan et al., 2016). It was reported that 82.6% of *V. parahaemolyticus* isolates were positive for PCR targeting the 16S rRNA, whereas in our study, 100% (Table 4) of *V. parahaemolyticus* isolates were identified as positive by PCR (Rojas et al., 2011). Our results for VPaI-2, VPaI-3, VPaI-6, Type I pilus, type III secretion systems (T3SS), and type VI secretion systems (T6SS) were consistent with those of other studies (Chao et al., 2009, 2010). The results of our study indicated that 100% of the *V. parahaemolyticus* isolates harbored the complete *L*-*tdh* genes (Table 4). Several studies revealed that 87.4, 93, and 86% of *V. parahaemolyticus* isolates carried the *tdh* gene (Bhoopong et al., 2007; Chen et al., 2016; Mizan et al., 2016). In the case of *trh* gene, all *V. parahaemolyticus* environmental isolates showed the negative amplification of PCR, and similar results were found in other study (Rojas et al., 2011). Recently, it was demonstrated that the virulence gene *trh* was absent in all *V. parahaemolyticus* isolates from water and mollusk (Silva et al., 2018). In another study, among 35 isolates from mussel only 4 isolates showed positive results against *trh* gene (Ottaviani et al., 2005). It was also reported that only 4 isolates were positive for *trh* virulence gene among 44 *V. parahaemolyticus* isolated from oysters (Kang et al., 2017). However, sometimes the presence or absence of virulence genes may depend on the differences in geographical regions, testing methodologies, and sample sources (Raghunath, 2015). For example, in a previous study, the *tdh* gene was detected in 20.7% of the seafood samples, from southwest coast of India by PCR after 18 h enrichment in ST broth. In the same study, it was isolated *tdh* carrying *V. parahaemolyticus* isolates from 19% of seafood samples, by colony hybridization following enrichment using ST broth (Raghunath, 2015), whereas, *tdh* was detected in 100% of the mussel samples from the west coast area of Korea by PCR after 24 h enrichment in LB broth in this study. It was reported that *tdh* gene was positive in 55 of environmental (water) samples, by MPN-PCR technique. But no *tdh* carrying strains were isolated by the conventional MPN-culture procedure (Alam et al., 2002).

In epidemiological research, REP-PCR is an effective and rapid typing method for the comparison and fingerprinting of *V. parahaemolyticus* isolates (Wong and Lin, 2001; Maluping et al., 2005). An earlier study using REP-PCR explained the intraspecific and interspecific differences between *V. parahaemolyticus* isolates and other strains (Jun et al., 2012). In our study, we obtained similar results using REP-PCR to compare and differentiating between intraspecific (10 *V. parahaemolyticus* isolates) and interspecific strains (*E. coli* ATCC43894, *V. harveyi* BB170) (Figure 1).

The environmental isolates in our study showed a high level of resistance to vancomycin, tetracycline, kanamycin, ampicillin, and penicillin (Table 5). Our findings were consistent with those of previous reports (Elexson et al., 2014a; Jiang et al., 2014; Xu et al., 2016; Kang et al., 2017; Tan et al., 2017; Ahmed et al., 2018). The high levels of multiple-antibiotic resistance property could be stated by the furthered chance to exchange genetic resistance determinants spotted on the plasmids among microorganisms, due to the extensive use of antibiotics in fishery and for the treatment of different kinds of infections (Ottaviani et al., 2013). It was demonstrated that the low MAR range (0.15) indicated low risk of contamination, whereas the high MAR range (above 0.25) indicated high risk of contamination (Chitanand et al., 2010). In this study, the higher MAR index values were 0.82, 0.73, and 0.64 (Table 6), indicating the high contamination ability of *V. parahaemolyticus* isolates; this agrees with the results obtained in other studies (Kang et al., 2017; Ahmed et al., 2018). However, appropriate monitoring is essential for developing a new generation of antibiotics and assuring the safety of seafood (Krumperman, 1983; Yu et al., 2016). The variation in the MAR index values may depend on sample sources (Tunung et al., 2010), geographic distribution (Lesley et al., 2011), and different testing methods (Robert-Pillot et al., 2004). It was described that the most possible source was cross-contamination from other products in the sampling location. However, cross-contamination could occur at any stage during the long processing and distribution chain, such as during pre-harvesting or post-harvest stage, contamination might occur through a contaminated container for transporting and improper handling (Chai et al., 2007). It was suggested that geographical locations and selective pressure influence the antibiotic resistance levels as well as multiple antibiotic resistance index. The high multiple antibiotic resistance value of *V. parahaemolyticus* isolates was 0.94, isolated from local cockles (*Anadara granosa*) obtained from a harvesting site in Tanjong Karang, Malaysia. The isolates were routinely grown at 35°C in LB broth with addition of 3% (w/v) NaCl (Lesley et al., 2011). In comparison with our study, the high multiple antibiotic resistance value of *V. parahaemolyticus* isolates was 0.82, isolated from local mussel (*M. coruscus*) from the west coast area of Korea, and grown at 30°C in LB broth with addition of 2% NaCl. Alternative strategies are urgently needed to overcome the continuous emergence of MDR in *V. parahaemolyticus* isolates in the environment (Tan et al., 2016).

Clinical strains and *V. parahaemolyticus* isolated from mussel possesses biofilm-forming abilities as well as pathogenic properties, and the relationship between the virulence genes detected with biofilm formation capabilities of the environmental isolates and clinical strains is variable in this study. The biofilm cell of *V. parahaemolyticus* has great resistant ability to antibiotics and disinfectants than planktonic cells (Song et al., 2017). The biofilm formation ability of *V. parahaemolyticus* increase the cells attachment ability to suspended particles, as well as shellfish (Elexson et al., 2014b). In this study, *V. parahaemolyticus* environmental isolates shown strong biofilm formation ability on both seafood surfaces at 30°C (Table 7). Previous study reported that *V. parahaemolyticus* environmental isolates produced strong biofilm on abiotic surface at 30°C (Mizan et al., 2016). The temperatures ranging 25–37°C was considered optimum for significant biofilm formation by *V. parahaemolyticus* isolates (Ahmed et al., 2018). There is limited study about biofilm formation on seafood. This study examined the biofilm formation ability of *V. parahaemolyticus* isolates on mussel surfaces for the first time. Interestingly, the result indicated that environmental isolates produced strong biofilm on mussel surface as well as shrimp surface (Table 7) than clinical isolates. Various studies described about better biofilm formation ability of environment isolates than clinical isolates (Kim B. R. et al., 2016; Qi et al., 2016). This might be due to differences in structural components such as pili, and fimbriac and adhesive surface proteins (Thompson et al., 2006; Abdallah et al., 2009). It could be also depended on various environmental conditions and bacterial adhesion properties (Wong et al., 2002; Sayen, 2014; Kim B. R. et al., 2016). Previous study reported *V. parahaemolyticus* biofilm formation ability on shrimp surface (Mizan et al., 2018), but no report was found on mussel surface. Therefore, further studies are needed to confirm the biofilm formation ability of environmental isolates on mussel surface. The SEM images showed the visual biofilm formation ability of both isolates on mussel (Figure 2A,B) and shrimp (Figure 2C,D) surface. Previous studies examined the SEM images of biofilm formation ability of *V. parahaemolyticus* on shrimp surface with different temperatures (Han et al., 2016). This study indicated that environmental isolates could produce significant biofilm on both surfaces and contaminate seafood, resulting in potential risk to consumer’s health.

## Conclusion

In this study, the tested *V. parahaemolyticus* isolates showed 100% positive amplification to pathogenic gene L-*tdh* and biofilm genes with strong genetic relationship. The mussel in the west coast area of Korea could be a positive source of resistance genes that may be transmitted to humans through consumption of mussel. The present study demonstrated that *V. parahaemolyticus* isolates carry the markers of different virulence genes, high antibiotic resistance profile and remarkable biofilm formation ability. These properties could be helpful to influence seafood contamination ability of these isolates significantly. This could be a good source of public health hazard, especially for seafood consumers. Therefore, monitoring of *V. parahaemolyticus* antibiotic resistance profile and others pathogenic factors are important to protect seafood in the marine environment and improve seafood safety in the seafood industry. The strong point of this study was the comprehensive coverage of genetic relationship, virulence factors as well as biofilm formation ability on seafood. Although only 8 environmental isolates were used in this study, the collected data are expected to be useful to build strong evidence in future research through increasing the number of isolates. This study recommends additional research using *V. parahaemolyticus* mussel isolates from different countries in the world and making a comprehensive statement about the variation of different virulence factors among the isolates of different countries.

## Author Contributions

MA, MM, HP, K-HB, NL, and SP provided assistance and guidance in throughout the research. MA wrote the manuscript. MM assisted the manuscript checking. All authors checked the manuscript and submitted final version.

## Conflict of Interest Statement

The authors declare that the research was conducted in the absence of any commercial or financial relationships that could be construed as a potential conflict of interest.

## Footnotes

1 https://www.msdsonline.com/resources/sds-resources/free-safety-data-sheet-index/Vibrio-parahaemolyticus/
